# Left Atrial Appendage Thrombus as a Marker of Disease Severity in 500 Patients with Atrial Fibrillation on Oral Anticoagulation: A 13-Year Follow-Up Study

**DOI:** 10.3390/jcm13175258

**Published:** 2024-09-05

**Authors:** Łukasz Turek, Marcin Sadowski, Jacek Kurzawski, Marianna Janion

**Affiliations:** Collegium Medicum, Jan Kochanowski University, 25-369 Kielce, Poland

**Keywords:** atrial fibrillation, anticoagulation, cardiovascular death, heart failure, left atrial appendage thrombus

## Abstract

**Background/Objective:** Whether left atrial appendage thrombus (LAAT) in patients with atrial fibrillation (AF) on chronic anticoagulation significantly increases cardiovascular risk is unknown. This study aimed to assess LAAT prevalence and its predictive role in cardiovascular events among consecutive anticoagulated patients with AF admitted for electrical cardioversion. **Methods:** This prospective study included 500 patients. The primary outcome was LAAT on transesophageal echocardiography. Patients were followed up for a median of 1927.5 (interquartile range 1004–2643) days to assess cardiovascular events. **Results:** LAAT was detected in 65 (13%) patients. No significant differences in stroke, transient ischemic attack, systemic thromboembolic events, or myocardial infarction prevalence were observed between patients with AF with and without LAAT. Hospitalization for heart failure (HF) was more frequent in patients with LAAT than in those without LAAT; however, the effect of LAAT on HF hospitalization was not statistically significant. Patients with LAAT had a significantly higher risk of cardiovascular death than those without LAAT. LAAT and greater left atrial (LA) diameter were associated with higher rates of cardiovascular death. The independent HF hospitalization predictors were greater LA diameter, lower left ventricular ejection fraction (LVEF), and estimated glomerular filtration rate (eGFR). **Conclusions:** Patients with AF who received anticoagulation therapy showed a high prevalence of LAAT. LAAT and greater LA diameter were associated with significantly higher rates of cardiovascular death. LAAT, greater LA diameter, lower LVEF, and lower eGFR were associated with poor prognosis in anticoagulated patients with AF and were predictors of disease severity.

## 1. Introduction

Atrial fibrillation (AF) is the most common arrhythmia in adults. The prevalence of AF is now estimated at 2%, double the rate reported in the last decade [1]. The risks of thrombus formation, stroke, heart failure (HF), and cardiovascular mortality are increased in patients with AF [2,3,4,5,6,7]. Recent therapeutic advances, including anticoagulation therapy, have led to a decrease in the incidence of stroke and systemic embolism in patients with AF. However, the rates of death and HF have remained unchanged over the years [8,9]. Studies have shown that HF following AF is associated with a higher risk of mortality [10,11]. Consequently, predicting hospitalization for HF in patients with AF is crucial for effective risk stratification in clinical practice. The left atrial appendage (LAA) is considered the most common site for thrombus formation in patients with AF [2,3,12]; however, it is unclear whether there is an association between left atrial appendage thrombus (LAAT) and health risks in patients with AF on chronic anticoagulation. The prevalence of LAAT in patients receiving appropriate oral anticoagulants (OACs) for direct-current cardioversion (DCC) remains uncertain. Most previous studies have focused on the short-term outcomes of different therapeutic strategies, usually during a follow-up period of 6–12 months [2]. Moreover, the role of clinical markers in predicting mortality, stroke, transient ischemic attack (TIA), systemic thromboembolic events, myocardial infarction, and hospitalization for HF in anticoagulated patients with AF during long-term follow-up remains poorly understood. Therefore, to better stratify the risk in patients with AF, we aimed to evaluate the association between LAAT and major adverse cardiovascular events in patients with anticoagulated AF during long-term follow-up.

## 2. Materials and Methods

### 2.1. Study Group

Consecutive adult patients on adequate OAC treatment with AF episodes lasting >48 h and poorly tolerated arrhythmia who were referred to the cardiology department for transesophageal echocardiography (TEE)-guided DCC for AF between December 2010 and March 2023 were included. Individuals with systolic blood pressure <90 mmHg, heart rate <60 beats per min, symptoms or signs of HF exacerbation, symptoms or signs of peripheral hypoperfusion, previous DCC, previous ablation, a prosthetic heart valve, moderate to severe mitral stenosis, determined as a mitral orifice area ≤1.5 cm^2^ and a mean pressure gradient of at least 5 mmHg, or previous intracardiac thrombus, were excluded. All data were prospectively collected from a database. Patients were followed up for at least 12 months from the day of TEE to assess the occurrence of mortality, stroke, TIA, systemic thromboembolic events, myocardial infarction, and HF hospitalization. Data regarding mortality, hospitalization for HF, and occurrence of stroke, TIA, systemic thromboembolic events, and myocardial infarction were obtained from patients and/or family members and the hospital database.

### 2.2. Anticoagulation Therapy

All patients on OAC treatment meeting the following criteria were eligible for the current trial: vitamin K antagonist (VKA) treatment with an international normalized ratio (INR) ≥2.0, or unbroken non-vitamin K antagonist oral anticoagulant (NOAC) for ≥3 weeks before enrolment in the trial [13,14,15,16]. Patients receiving VKA had their INR levels monitored weekly for 3 weeks, ensuring all results remained within the therapeutic range. Patients prescribed apixaban were required to take a dose of 5 mg orally twice daily, although a reduced dose of 2.5 mg twice daily was permitted for those meeting at least two of the following criteria: age ≥ 80 years, body weight ≤ 60 kg, or serum creatinine level ≥ 1.5 mg/dL. Patients prescribed dabigatran were instructed to take a dose of 150 mg orally twice daily as the standard dose. However, individuals with at least one of the following additional characteristics were permitted to take a reduced dose of 110 mg orally twice daily: HAS-BLED score ≥ 3, age ≥ 80 years, estimated glomerular filtration rate (eGFR) of 30–49 mL/min/1.73 m^2^, or concurrent treatment with verapamil [14]. Patients prescribed rivaroxaban were required to take a dose of 20 mg orally once daily as the standard dose. However, a reduced dose of 15 mg orally once daily was permitted for individuals meeting at least one of the following criteria: HAS-BLED score ≥ 3 and/or eGFR of 15–49 mL/min/1.73 m^2^. The eGFR was obtained using the Modification of Diet in Renal Disease (MDRD) glomerular filtration rate equation [17]. The first patient anticoagulated with VKAs was recruited on 14 December 2010. The first patient was enrolled in the Dabigatran study on 6 March 2013, followed by Rivaroxaban on 6 October 2015, and Apixaban on 7 November 2016.

### 2.3. Echocardiographic Examination

Each examination was conducted by an experienced, certified echocardiographer using Vivid E9 and Vivid E95 cardiovascular ultrasound systems (GE Vingmed Ultrasound AS, Horten, Norway), equipped with a multiplanar ultrasonic probe. All data collection adhered strictly to the established protocol [18]. The LAA characteristics were obtained on TEE, using multiplanar imaging in the mid-esophageal view with an adequate depth and gain. The thrombus was identified as a uniformly echo-dense intracavitary structure with distinct borders distinguishable from the endocardium. This structure was consistently observed throughout all phases of the cardiac cycle, evident in at least two imaging planes, and was not associated with the pectinate muscles [19,20]. The anteroposterior diameter of the left atrium (LA) was measured during end-systole in a plane perpendicular to the long axis of the ascending aorta, using the parasternal long-axis view. The left ventricular ejection fraction (LVEF) was evaluated using Simpson’s biplane method [21]. The findings from each TEE and transthoracic echocardiogram were documented, stored, and accessible for further review as necessary.

### 2.4. Statistical Analysis

Continuous data are presented as means, standard deviations, medians, and quartiles. Categorical data are summarized as frequencies and percentages. The normality of the distributions was checked using the Shapiro–Wilk test. Owing to the non-normality of all continuous data, the Mann–Whitney test was used to compare groups according to LAAT status, whereas the chi-squared or Fisher’s exact test was applied for categorical variables. Hazard ratios (HR) with 95% confidence intervals (CIs) were calculated, and univariable and multivariable analyses of the potential predictors of cardiovascular death and HF hospitalization were performed using Cox proportional hazard models. Considering cardiovascular death as the endpoint, the Kaplan–Meier method was applied to create survival curves, and the log-rank test was used to compare survival according to LAAT status. Statistical significance was set at a two-tailed *p*-value < 0.05. All statistical analyses were executed using the R software package, version 4.0.3 (R Foundation for Statistical Computing, Vienna, Austria). 

The study adhered to the principles outlined in the Declaration of Helsinki and received approval from the local ethics committee (Approval No.: 21/2010). Informed consent was obtained from all participants involved in the study.

## 3. Results

Overall, 500 consecutive patients with AF who received OAC treatment were enrolled in this prospective study. Of these, 418 (83.6%) underwent DCC, with conversion to sinus rhythm occurring in 365 (87.3%) patients. Of the 82 patients disqualified from DCC, 65 (13%) had LAAT, and the remaining patients had multiple prior relapses of arrhythmia and were offered control of the heart rate. No periprocedural complications were observed. Thrombi were detected only in the LAA on TEE.

The baseline characteristics according to LAAT status are presented in Table 1. On average, patients with LAAT were older, had a lower body mass index, and had higher CHA_2_DS_2_-VASc scores. They were more likely to have HF, previous myocardial infarction, greater LA diameter, and lower LVEF, and to be on anticoagulation with VKA. 

The study population was followed up on for a median of 1927.5 (interquartile range 1004–2643) days to assess the survival rate and incidence of stroke, TIA, systemic thromboembolic events, myocardial infarction, and HF hospitalization. During follow-up, overall and cardiovascular mortality, as well as HF hospitalization rates, were significantly higher in patients with LAAT. Other cardiovascular events occurred at a similar rate in patients with AF with and without LAAT (Table 2). 

**Figure 1:**
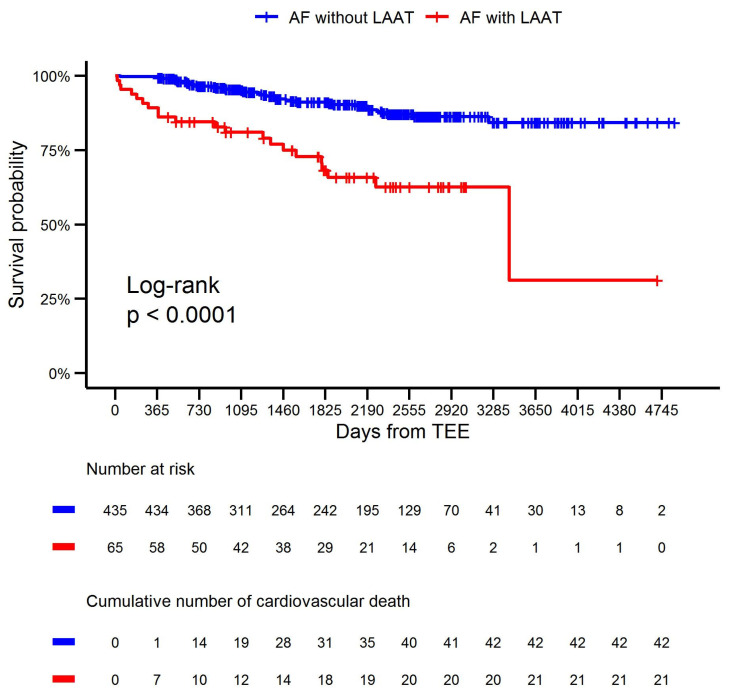
Kaplan–Meier survival curves and log-rank tests were used to compare the days to cardiovascular death in the cohorts of AF without LAAT and AF with LAAT. Abbreviations: AF, atrial fibrillation; LAAT, left atrial appendage thrombus; and TEE, transesophageal echocardiography.

The Kaplan–Meier curves for cardiovascular survival in the LAAT and non-LAAT groups are presented in Figure 1.

Statistical analysis confirmed that LAAT was associated with a higher rate of cardiovascular death (HR: 2.03, 95% CI: 1.13–3.65, *p* = 0.02). Moreover, a greater LA diameter was associated with a higher rate of cardiovascular death (HR: 1.09, 95% CI: 1.03–1.14, *p* = 0.003) and HF hospitalization (HR: 1.11, 95% CI: 1.06–1.16, *p* < 0.001). Furthermore, a lower LVEF (HR: 0.97, 95% CI: 0.95–0.99, *p* = 0.005) and lower eGFR (HR: 0.97, 95% CI: 0.96–0.99, *p* = 0.007) were associated with a higher rate of HF hospitalization. In a multivariable model that considered additional variables, LAAT was not associated with a higher rate of HF hospitalization (HR: 1.67, 95% CI: 0.94–2.98, *p* = 0.08). Moreover, the predictive value of anticoagulant agent type during long-term follow-up was not confirmed (Table 3 and Table 4).

## 4. Discussion

The main finding of our study was that although patients with AF were on anticoagulation therapy according to the guidelines, the rate of LAAT was high, reaching 13%. The presence of LAAT and greater LA diameter were independent predictors of cardiovascular mortality.

Interestingly, anticoagulation with VKA was more frequently observed in the LAAT group; however, the predictive value of anticoagulant agent type during long-term follow-up was not confirmed. It remains unclear whether the presence of LAAT reliably indicates the adequacy of NOACs compared to VKAs in patients with AF [1]. Recently, the use of NOACs has become increasingly widespread. Our study revealed a temporal trend in anticoagulation treatment, with a decreasing proportion of patients receiving VKAs compared to those receiving NOACs. In recent years, very few patients have been treated with VKAs. The effectiveness and safety of various NOACs have been extensively studied and compared with those of VKAs in different clinical settings. Based on high-quality evidence, NOACs are now recommended over VKAs to reduce the risk of mortality, stroke, systemic embolism, and intracranial hemorrhage [2,3]. High-quality evidence supports the use of OACs to reduce the risk of ischemic stroke in patients with AF. However, long-term OAC therapy may be contraindicated in certain cases, such as when spontaneous intracranial bleeding occurs due to an irreversible cause. Percutaneous left atrial appendage occlusion is a reasonable alternative for stroke prevention in these patients [2,22].

In our study, a lower BMI was more frequently observed in the LAAT group; however, the predictive value of BMI during long-term follow-up has not yet been established. It remains unclear whether a lower BMI significantly increases the risk of thrombogenesis in patients with AF receiving OACs. However, this hypothesis requires further investigation. Although the correlation between obesity and the development of AF is well documented, little is known about its impact on therapeutic interventions and outcomes, including thrombus formation in individuals already affected by AF [23]. Data on the impact of obesity on clinical outcomes in individuals with AF are inconsistent [24]. Some studies suggest that a higher BMI may be associated with more favorable clinical outcomes in AF and other cardiovascular diseases, a phenomenon referred to as the “obesity paradox” [24]. However, other studies present a contradictory view, indicating that overweight or obese individuals with AF may not have a better prognosis [24]. 

Moreover, LAAT plays a predictive role in risk stratification for cardiovascular death in anticoagulated patients with AF. However, the predictive value of LAAT for hospitalization due to HF was not confirmed, and the rates of stroke, TIA, systemic thromboembolic events, and myocardial infarction were relatively low and similar across all LAAT status groups. Additionally, another imaging biomarker, greater LA diameter, was identified as a predictor of cardiovascular mortality and HF hospitalization, and lower LVEF had a predictive value for HF hospitalization, similar to lower eGFR. In accordance with the recommendations for AF provided by the American Heart Association and European Society of Cardiology [2,3], LAAT detection prior to cardioversion influences the decision-making process. If we were to strictly rely on our findings related to the high rate of LAAT, we would suggest paying more attention and applying more caution when managing patients at high risk of LAAT who are referred for cardioversion without TEE guidance. However, in a study by Frederiksen et al., which evaluated 2150 patients with AF receiving OAC therapy, the thromboembolic complication rate for non-TEE-guided cardioversions was low, and thromboembolism occurred in one of 684 patients (0.15%) receiving NOAC therapy and in two of 1466 patients (0.14%) taking warfarin [25]. Moreover, in a study by Klein et al., which evaluated 1222 patients with AF and an arrhythmia duration of >2 days, no significant difference was observed in the embolic event rate between TEE-guided cardioversion and the conventional approach of 3 weeks of warfarin treatment before cardioversion (0.8% vs. 0.5%, respectively; *p* = 0.5) [26]. Therefore, we speculate that DCC after 3 weeks of effective anticoagulation carries the same risk of thromboembolism irrespective of LAAT status on TEE; hence, further research is suggested.

LAAT is considered a risk factor for stroke in patients with AF [3,27,28,29]; however, the predictive value of LAAT for mortality and cardiovascular morbidity in patients on OAC therapy remains unknown. In a study by Schaeffer et al., the overall prevalence of thrombi was 4.7%. Patients not receiving anticoagulant treatment had the highest prevalence (9.5%), whereas the thrombus rate was lower (4.1%) among those who were anticoagulated. Nevertheless, this study did not include a follow-up period [30]. In a study of 424 Korean patients, thrombus was found in 2.2% of patients on VKA examined before DCC, compared to 4.3% on NOAC (*p* = 0.28); however, this study did not have a follow-up period [31]. In a study of 510 Italian patients with persistent AF scheduled for TEE-guided DCC, the rate of LAAT was exceptionally low, reaching 0.6% in those treated with VKA and 0.6% in those treated with dabigatran; however, all patients with LAAT were excluded from the follow-up part of the study [32]. In a study by Frenkel et al., thrombus was observed in 4.4% of patients taking NOACs who were scheduled for catheter ablation of AF and atrial flutter, which was comparable to the findings in the warfarin group; however, this study did not include a follow-up period [33]. In a retrospective study by Gorczyca et al., which evaluated 1256 patients with AF taking rivaroxaban or dabigatran, the thrombus detection rate was 4.1% on TEE before catheter ablation or DCC, regardless of the anticoagulant type; however, the study was not designed with a follow-up evaluation [34]. In a study by Angelini et al., TEE was performed in 352 consecutive patients with AF receiving NOACs, and thrombi were detected in 27 (7.7%) patients; however, the study did not involve any follow-up [35]. Recently, we examined 296 patients with AF on OAC treatment referred for DCC, and a high prevalence of LAAT, reaching 14.5%, was observed, with no difference between the different types of OACs (*p* = 0.26). Moreover, no strokes or systemic thromboembolic events occurred during the 12 months following TEE, but there were three deaths (1.01%). All deaths occurred in the heart rate control group in patients with HF with reduced ejection fraction and LAAT; however, these deaths were not considered LAAT-related [1]. In a prospective study by Durmaz et al., among 184 patients with AF receiving OAC treatment, 28 (15.2%) had LA or LAA thrombi on TEE, and the patients were followed up for a median of 12 (7–20) months. There was a significant association between thrombi and ischemic stroke, and patients with thrombi experienced ischemic stroke more frequently than those without thrombi (7.1% vs. 4.4%, *p* = 0.001, respectively). However, there was no significant relationship with regard to cardiac and non-cardiac mortality between these groups [36]. In a study by Nair et al., among 226 consecutive patients with AF, 95 had thrombus on TEE, including 91 located in the LAA and four localized in the body of the LA. However, the included patients were either on untherapeutic anticoagulation or not on anticoagulation; subsequently, unless warfarin was contraindicated, anticoagulation with warfarin was started for patients who were not on warfarin or was intensified for patients with a subtherapeutic INR. Seven of the ninety-five patients (7%) with thrombus experienced a new cerebrovascular event (stroke in six patients and TIA in one patient) during follow-up. Five of the one hundred thirty-one non-thrombus patients (4%) experienced a new cerebrovascular event (stroke in one patient and TIA in four patients) during follow-up (*p* not significant). Thirty-three of the ninety-five patients with thrombi (35%) died during the follow-up period. Nineteen of the one hundred thirty-one patients without thrombi (15%) died during the follow-up period. Survival rates were significantly higher in patients without thrombus than in those with thrombus (*p* < 0.001) [37]. In a secondary post hoc analysis of the ARISTOTLE trial conducted by Vinereanu et al., 127 of 1251 patients with AF who were anticoagulated with apixaban or warfarin had LA/LAA thrombi. The rate of stroke/systemic embolism was not significantly different between patients with and without LA/LAA thrombus (HR: 1.27, 95% CI: 0.23–6.86). The rates of ischemic stroke, myocardial infarction, cardiovascular death, and all-cause death did not differ between patients with and without LA/LAA thrombus [38]. In a prospective study by Kosmalska et al., of 267 patients scheduled for DCC due to persistent AF/atrial flutter, 77 (29%) were diagnosed with thrombus or sludge. Some patients did not receive anticoagulation therapy. The annual mortality rate of patients with thrombi or sludge was 23%. The annual mortality rate in the group without thrombi was 1.6%. Overall, 17% of patients with thrombus or sludge experienced ischemic stroke. In patients without thrombi, the risk of stroke is 1% [39].

Based on the available research, data regarding anticoagulated patients with AF and the predictive role of LAAT for cardiovascular morbidity and mortality are limited, and further research is warranted.

Our study also provides insights into the effects of various biomarkers and comorbidities on the outcomes of patients with AF undergoing anticoagulation therapy, contributing to the more nuanced characteristics of these patients. We identified that greater LA diameter, lower LVEF, and lower eGFR were prognostic factors for poor outcomes in anticoagulated patients with AF. We speculate that they may be considered markers of disease severity. Additionally, a study by Hamatani et al. found that a larger anteroposterior LA diameter was significantly associated with an increased risk of hospitalization for heart failure in AF patients with preserved LVEF [8]. In a study by Taniguchi et al., which included 422 patients with AF, 52 patients (12.3%) developed at least one new HF event during the follow-up period. Significant independent predictors of new HF events were advancing age, lower LVEF, higher indexed left ventricular mass, and larger indexed LA volume [9]. In a study by Hamatani et al., a lower LVEF was significantly associated with a higher risk of hospitalization for HF in AF patients without pre-existing HF [40]. Santhanakrishnan et al. found that the presence of both AF and HF was associated with a greater risk of mortality, particularly among individuals with new HF and reduced ejection fraction [41]. Kotecha et al. reported that all-cause mortality was significantly higher in AF patients with HF and reduced ejection fraction than in those with preserved ejection fraction, although the risks of stroke and HF hospitalization were similar between the two groups [42]. A prospective study conducted in Japan, which included 1942 patients with AF, found that anemia, renal dysfunction (eGFR <60 mL/min/m^2^), diabetes mellitus, and organic heart disease were independently associated with the incidence of HF [43]. Additionally, a study by Potpara et al. identified that a history of diabetes mellitus, a history of hypertension, a dilated left atrium, and low-normal LVEF (50–54%) were significant predictors of subsequent HF (all *p* < 0.05) [44]. 

Data on the predictive roles of LA size, eGFR, and LVEF in cardiovascular events in anticoagulated patients with AF are limited, and further research is required. However, these parameters have been studied more extensively in relation to cardiovascular events than LAAT.

Following the current recommendations for AF, appropriate and complex management of comorbidities is required [2,3,4,45].

### Limitations

First, the sample size was relatively small. There was a lack of patients treated with edoxaban. Echocardiographic contrast agents were not used to improve echocardiographic imaging; therefore, overdiagnosis of LAAT cannot be ruled out. The MDRD equation was used to estimate the glomerular filtration rate. However, this equation indexes the glomerular filtration rate to a body surface area of 1.73 m^2^ and is not adjusted for the actual body surface area, which we did not evaluate; therefore, dabigatran and rivaroxaban dosing may not have been optimal. All patients receiving NOAC therapy reported uninterrupted anticoagulation treatment; however, compliance could not be verified alternatively. Nonetheless, all study participants demonstrated satisfactory awareness and knowledge of anticoagulation treatment. Finally, we did not assess the total duration of OAC treatment or the total duration of arrhythmia before admission and enrolment. Moreover, we did not assess the left atrial area, left atrial volume index, LAA morphology, or emptying velocity.

## 5. Conclusions

In our study, the presence of LAAT, greater left atrial diameter, lower LVEF, and lower eGFR emerged as significant markers of poor prognosis in anticoagulated patients with AF. These findings highlight the importance of comprehensive clinical assessment in this population, as these markers can provide valuable insights into disease severity and the risk of adverse outcomes. The inclusion of LAAT presence in risk stratification models might facilitate more personalized and timely therapeutic interventions, such as continuous dosage optimization and drug selection. Our findings suggest that clinicians may consider closer monitoring and potentially more aggressive management strategies for patients with these risk factors. 

Furthermore, our study paves the way for future research to explore targeted therapies (e.g., strict monitoring and more aggressive therapeutic interventions for comorbidities) that could mitigate the heightened risks associated with these prognostic indicators. Longitudinal studies and trials focusing on intervention strategies that address these specific markers may offer new avenues for improving outcomes in this high-risk patient group.

## Figures and Tables

**Table 1:** Baseline characteristics of patients with atrial fibrillation and with or without left atrial appendage thrombus.

Variable	Patients with LAAT (*n* = 65)	Patients without LAAT (*n* = 435)	*p* Value
Female sex	26 (40)	172 (39.5)	0.94
Age, years	68.4 (8.9)	64.0 (9.8)	0.001
BMI, kg/m^2^	28.2 (26.0–32.4)	29.4 (27.1–32.9)	0.045
CHA_2_DS_2_-VASc score	4 (3–4)	3 (2–4)	<0.001
Heart rate, 1/min	100 (85–115)	90 (80–110)	0.1
SBP, mmHg	130 (120–130)	125 (120–130)	0.67
DBP, mmHg	80 (70–80)	80 (70–80)	0.4
eGFR, mL/min/1.73 m^2^	66.1 (50.3–75.3)	66.9 (57.9–76.4)	0.09
LA diameter, mm	47 (44–52)	44 (42–48)	<0.001
LVEF, %	46 (30–56)	59 (50–60)	<0.001
Previous stroke/TIA/systemic thromboembolism	5 (7.7)	33 (7.6)	>0.99
Arterial hypertension	50 (76.9)	338 (77.7)	0.89
HF	49 (75.4)	223 (51.3)	<0.001
Diabetes mellitus	14 (21.5)	92 (21.1)	0.94
COPD	6 (9.2)	18 (4.1)	0.11
Previous MI	10 (15.4)	28 (6.4)	0.02
PAD or aortic plaque	9 (13.8)	30 (6.9)	0.051
Anticoagulation on admission			
VKA	14 (21.5)	50 (11.5)	0.02
Rivaroxaban	18 (27.7)	127 (29.2)	0.8
Apixaban	6 (9.2)	67 (15.4)	0.19
Dabigatran	27 (41.5)	191 (43.9)	0.72
Medication at discharge			
VKA	17 (26.2)	46 (10.6)	<0.001
Rivaroxaban	10 (15.4)	129 (29.7)	0.02
Apixaban	13 (20.0)	69 (15.9)	0.4
Dabigatran	25 (38.5)	191 (43.9)	0.41
ACEI or ARB	49 (75.4)	316 (72.6)	0.64
Beta-blocker	62 (95.4)	386 (88.7)	0.1
Statin	40 (61.5)	275 (63.2)	0.79

Data are presented as numbers (percentages), means (standard deviations), or medians (interquartile ranges), as appropriate. Abbreviations: ACEI, angiotensin-converting-enzyme inhibitors; ARB, angiotensin II receptor blockers; BMI, body mass index; CHA_2_DS_2_-VASc, scale for stroke and thromboembolic risk assessment; COPD, chronic obstructive pulmonary disease; DBP, diastolic blood pressure; eGFR, estimated glomerular filtration rate; HF, heart failure; LA, left atrium; LAAT, left atrial appendage thrombus; LVEF, left ventricular ejection fraction; MI, myocardial infarction; PAD, peripheral artery disease; SBP, systolic blood pressure; TIA, transient ischemic attack; and VKA, vitamin K antagonists.

**Table 2:** Follow-up outcomes in patients with atrial fibrillation and with or without left atrial appendage thrombus.

Variable	Patients with LAAT (*n* = 65)	Patients without LAAT (*n* = 435)	*p* Value
HF hospitalization	23 (35.4)	49 (11.3)	<0.001
MI	3 (4.6)	8 (1.8)	0.16
Systemic thromboembolism	1 (1.5)	2 (0.5)	0.34
TIA	1 (1.5)	4 (0.9)	0.503
Stroke	3 (4.6)	10 (2.3)	0.23
Cardiovascular death	21 (32.3)	42 (9.7)	<0.001
All-cause death	23 (35.4)	48 (11)	<0.001

Data are presented as numbers (percentages). Abbreviations: HF, heart failure; LAAT, left atrial appendage thrombus; MI, myocardial infarction; and TIA, transient ischemic attack.

**Table 3:** Cox proportional hazard regression analysis with potential predictors of cardiovascular death.

Variable	Univariable HR (95% CI)	*p* Value	Multivariable HR (95% CI)	*p* Value
LAAT	3.9 (2.31–6.59)	<0.001	2.03 (1.13–3.65)	0.02
Male sex	1.2 (0.71–2.01)	0.49		
Age, year	1.05 (1.02–1.09)	<0.001	1.02 (0.98–1.06)	0.41
BMI, kg/m^2^	0.97 (0.92–1.03)	0.29		
CHA_2_DS_2_-VASc score per 1	1.37 (1.17–1.6)	<0.001	1.09 (0.86–1.38)	0.47
Heart rate, 1/min	1 (0.99–1.01)	0.98		
SBP, mmHg	0.98 (0.96–1.01)	0.07		
DBP, mmHg	0.98 (0.95–1.01)	0.22		
eGFR, mL/min/1.73 m^2^	0.97 (0.95–0.98)	<0.001	0.98 (0.96–1)	0.051
LA diameter, mm	1.13 (1.08–1.18)	<0.001	1.09 (1.03–1.14)	0.003
LVEF, %	0.95 (0.94–0.97)	<0.001	0.99 (0.97–1.01)	0.29
Previous stroke, TIA, systemic thromboembolism	1.58 (0.72–3.48)	0.25		
Arterial hypertension	0.91 (0.51–1.63)	0.76		
HF	2.56 (1.5–4.25)	<0.001	1.15 (0.6–2.21)	0.67
Diabetes mellitus	1.38 (0.77–2.46)	0.28		
COPD	1.48 (0.54–4.08)	0.45		
Previous MI	2.54 (1.29–4.99)	0.01	1.72 (0.85–3.47)	0.13
PAD or aortic plaque	1.84 (0.87–3.86)	0.11		
Anticoagulation on admission				
VKA	1.84 (1.05–3.24)	0.03		
Rivaroxaban	0.76 (0.42–1.39)	0.37		
Apixaban	0.66 (0.24–1.85)	0.43		
Dabigatran	0.91 (0.55–1.5)	0.72		
Medication at discharge				
VKA	1.85 (1.05–3.25)	0.03	1.45 (0.81–2.58)	0.21
Rivaroxaban	0.64 (0.34–1.21)	0.17		
Apixaban	0.71 (0.28–1.76)	0.47		
Dabigatran	1.01 (0.62–1.66)	0.96		
ACEI or ARB	1.09 (0.62–1.93)	0.76		
Beta-blocker	2.45 (0.77–7.81)	0.13		
Statin	0.79 (0.48–1.32)	0.37		

Abbreviations: ACEI, angiotensin-converting-enzyme inhibitors; ARB, angiotensin II receptor blockers; BMI, body mass index; CHA_2_DS_2_-VASc, scale for stroke and thromboembolic risk assessment; CI, confidence interval; COPD, chronic obstructive pulmonary disease; DBP, diastolic blood pressure; eGFR, estimated glomerular filtration rate; HF, heart failure; HR, hazard ratio; LA, left atrium; LAAT, left atrial appendage thrombus; LVEF, left ventricular ejection fraction; MI, myocardial infarction; PAD, peripheral artery disease; SBP, systolic blood pressure; TIA, transient ischemic attack; and VKA, vitamin K antagonists.

**Table 4:** Cox proportional-hazards regression analysis with potential predictors of heart failure hospitalization.

Variable	Univariable HR (95% CI)	*p* Value	Multivariable HR (95% CI)	*p* Value
LAAT	3.99 (2.42–6.58)	<0.001	1.67 (0.94–2.98)	0.08
Male sex	1.14 (0.7–1.84)	0.6		
Age, year	1.05 (1.02–1.08)	0.001	1.01 (0.97–1.05)	0.62
BMI, kg/m^2^	1.01 (0.96–1.06)	0.81		
CHA_2_DS_2_-VASc score per 1	1.38 (1.19–1.59)	<0.001	1.15 (0.91–1.45)	0.23
Heart rate, 1/min	1.02 (1.01–1.03)	0.002	1.01 (0.99–1.02)	0.07
SBP, mmHg	0.99 (0.98–1.02)	0.89		
DBP, mmHg	1.01 (0.98–1.05)	0.4		
eGFR, mL/min/1.73 m^2^	0.96 (0.94–0.97)	<0.001	0.97(0.96–0.99)	0.007
LA diameter, mm	1.16 (1.12–1.21)	<0.001	1.11 (1.06–1.16)	<0.001
LVEF, %	0.94 (0.93–0.95)	<0.001	0.97 (0.95–0.99)	0.005
Previous stroke, TIA, systemic thromboembolism	1.59 (0.76–3.33)	0.21		
Arterial hypertension	1.36 (0.75–2.49)	0.31		
HF	2.43 (1.48–3.99)	<0.001	0.79 (0.42–1.51)	0.48
Diabetes mellitus	1.54 (0.91–2.62)	0.1		
COPD	1.25 (0.46–3.43)	0.66		
Previous MI	1.85 (0.92–3.72)	0.08		
PAD or aortic plaque	2.02 (1.01–4.08)	0.048	1.23 (0.56–2.71)	0.61
Anticoagulation on admission				
VKA	2.18 (1.28–3.7)	0.004		
Rivaroxaban	0.89 0.52–1.52)	0.66		
Apixaban	0.85 (0.38–1.86)	0.68		
Dabigatran	0.71 (0.44–1.14)	0.15		
Medication at discharge				
VKA	1.86 (1.07–3.22)	0.03	1.34 (0.76–2.35)	0.31
Rivaroxaban	1.01 (0.59–1.7)	0.99		
Apixaban	1.11 (0.56–2.18)	0.77		
Dabigatran	0.64 (0.39–1.05)	0.07		
ACEI or ARB	1.23 (0.71–2.12)	0.45		
Beta-blocker	3.02 (0.95–9.64)	0.06		
Statin	0.95 (0.59–1.53)	0.83		

Abbreviations: ACEI, angiotensin-converting-enzyme inhibitors; ARB, angiotensin II receptor blockers; BMI, body mass index; CHA_2_DS_2_-VASc, scale for stroke and thromboembolic risk assessment; CI, confidence interval; COPD, chronic obstructive pulmonary disease; DBP, diastolic blood pressure; eGFR, estimated glomerular filtration rate; HF, heart failure; HR, hazard ratio; LA, left atrium; LAAT, left atrial appendage thrombus; LVEF, left ventricular ejection fraction; MI, myocardial infarction; PAD, peripheral artery disease; SBP, systolic blood pressure; TIA, transient ischemic attack; and VKA, vitamin K antagonists.

## Data Availability

The data that support the findings of this study are available from the corresponding author, Ł.T., upon reasonable request.
